# Evaluation of the Fetal Brain in Maternal Hypothyroidism and Thyroid Autoantibody Positivity: A Prospective Case-Control Study

**DOI:** 10.3390/jcm14072204

**Published:** 2025-03-24

**Authors:** Raziye Torun, Hakan Golbasi, Ceren Saglam, Sevim Tuncer Can, Ilayda Gercik, Hale Ankara Aktas, Ilknur Toka, Zubeyde Emiralioglu Cakir, Mustafa Sengul, Atalay Ekin

**Affiliations:** 1 Department of Perinatology, Izmir City Hospital, 35510 Izmir, Turkey; drhkngolbasi@gmail.com (H.G.); drcerensaglam@yahoo.com (C.S.); drsevimtuncer@hotmail.com (S.T.C.); ilgercik@gmail.com (I.G.); haleankara@gmail.com (H.A.A.); drilknurtoka@gmail.com (I.T.); zubeydeemiralioglu@hotmail.com (Z.E.C.); atalayekin@hotmail.com (A.E.); 2 Department of Obstetrics and Gynecology, Faculty of Medicine, Izmir Katip Celebi University, 35620 Izmir, Turkey; dr.mustafasengul@gmail.com

**Keywords:** fetal brain, maternal hypothyroidism, thyroid antibody positivity, prenatal ultrasonography

## Abstract

**Background/Objectives**: Maternal thyroid function plays a crucial role in fetal brain development, yet the potential impact of maternal hypothyroidism and thyroid autoimmunity on fetal intracranial structures remains inadequately explored. To investigate the impact of maternal hypothyroidism and thyroid autoimmunity on fetal intracranial structures, focusing on potential alterations in critical brain parameters during mid-gestation. **Methods**: This prospective case-control study included pregnant women between 18 and 24 weeks of gestation. Participants were divided into three groups: hypothyroidism and antibodies (Ab) group, hypothyroidism and Ab(–) group, and the control group. Ultrasonographic measurements of fetal intracranial structures such as the posterior lateral ventricle (PLV), cavum septum pellucidi (CSP), cisterna magna (CM), thalamus, and transcerebellar diameter (TCD) were recorded and compared. **Results**: A total of 153 pregnant women were evaluated (n = 52 in the hypothyroidism and Ab(+) group, n = 51 in the hypothyroidism and Ab(−) group, and n = 50 in the control group). Although most of the biometric parameters were similar across the groups, the hypothyroidism and Ab(+) group exhibited significantly lower PLV and thalamus measurements compared to the control group (*p* < 0.05). Additionally, there was a notable difference in the BMI among the groups, with hypothyroid participants (with or without antibodies) showing higher rates of being overweight or obese. **Conclusions**: Maternal hypothyroidism and the presence of thyroid autoantibodies may be associated with subtle changes in fetal brain structures during the mid-gestation period, particularly in the thalamus and PLV.

## 1. Introduction

Thyroid dysfunction is one of the most common endocrine disorders among women of reproductive age [1]. Because the fetus only begins producing thyroid hormones between the 16th and 20th weeks of gestation, the thyroid hormones necessary for the optimal growth and development of various organ systems, particularly the fetal brain, are predominantly supplied by transplacental transfer from the mother, starting as early as the first trimester [2]. Hashimoto’s thyroiditis, an autoimmune disease, is the most common cause of hypothyroidism and has a prevalence of approximately 8–10% among women of reproductive age [3]. Characterized by the presence of anti-thyroid antibodies, this condition can lead to the transplacental passage of thyroid autoantibodies, potentially suppressing neonatal thyroid function. However, such suppression is typically transient, and there is insufficient evidence of any long-term adverse effects on the infant [4,5]. Thyroid autoimmunity may alter the course and outcome of pregnancy [6,7,8]. It is thought that anti-thyroid-antibody positivity, even in the presence of a euthyroid state, reflects a broader autoimmune imbalance that can give rise to increased pregnancy complications [9].

The adverse effects of hypothyroidism during pregnancy are well documented and include associated fetal and neonatal complications such as preterm birth, low birth weight, intrauterine fetal death, increased incidence of neonatal respiratory distress, and neurodevelopmental dysfunction in the infant [5,10,11,12,13,14,15,16]. Maternal hypothyroidism during pregnancy is defined as an elevated thyroid-stimulating hormone (TSH) concentration exceeding the upper limit of the gestation-specific reference range [17]. Previous research also suggests that thyroid peroxidase antibodies (TPOAb) may negatively affect pregnancy outcomes in euthyroid women, although the precise mechanism remains a subject of debate.

While various fetal and maternal complications have been documented in pregnancies complicated by thyroid autoantibodies or hypothyroidism, no study to date has focused specifically on fetal intracranial development and related changes. Therefore, in this study, we aimed to evaluate the fetal intracranial structures sonographically in hypothyroid pregnant women, considering both the presence of autoantibodies and the need for treatment.

## 2. Materials and Methods

This prospective case-control study was conducted between June and December 2024 at the Perinatology Clinic of İzmir City Hospital. Ethical approval for the study was obtained from the ethics committee of Izmir City Hospital (approval no: 2024/51). All of the pregnant women participating in the study were informed about the research, and detailed informed consent was obtained from each participant.

Patients presenting to our clinic between 18 and 24 weeks of gestation for fetal anomaly screening were included in the study. Participants were divided into three groups, namely hypothyroidism and antibodies (Ab)[+]): pregnant women with hypothyroidism secondary to Hashimoto’s thyroiditis diagnosed prior to pregnancy, receiving levothyroxine therapy, and testing positive for TPOAb and thyroglobulin antibodies (TGAb); hypothyroidism and Ab[−]: pregnant women without a history of hypothyroidism, who had negative TPOAb and TGAb levels but had TSH > 2.5 mIU/L in the first trimester and subsequently received levothyroxine therapy; and the control group: pregnant women with no hypothyroidism or thyroid autoantibodies.

The presence of TPOAb and TGAb was determined using enzyme-linked immunosorbent assay (ELISA; ORGENTEC, Mainz, Germany) according to the manufacturer’s instructions. The cutoff value for TPOAb positivity was >34 IU/mL, and for TGAb positivity was >40 IU/mL.

Exclusion criteria were multiple gestations; pregnancies complicated by fetal malformations or chromosomal anomalies; a history of thyroid surgery, thyroid nodules, or radioactive iodine therapy; and the presence of additional systemic diseases.

**Figure 1:**
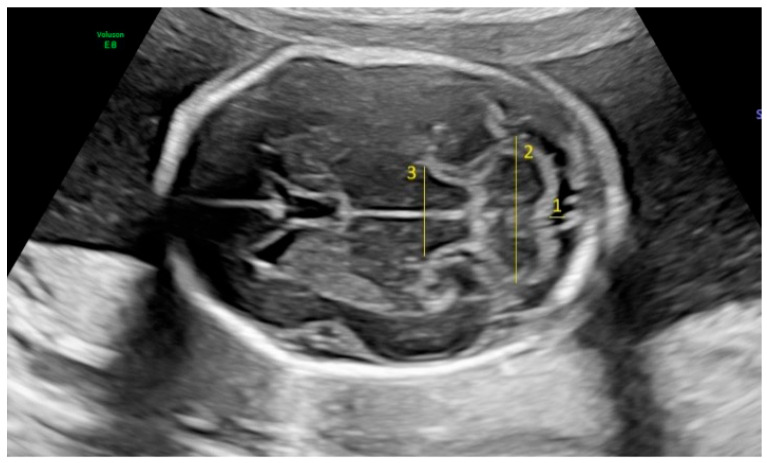
Fetal intracranial structures in the transcerebellar plane (1: cisterna magna; 2: cerebellum; 3: thalamus).

**Figure 2:**
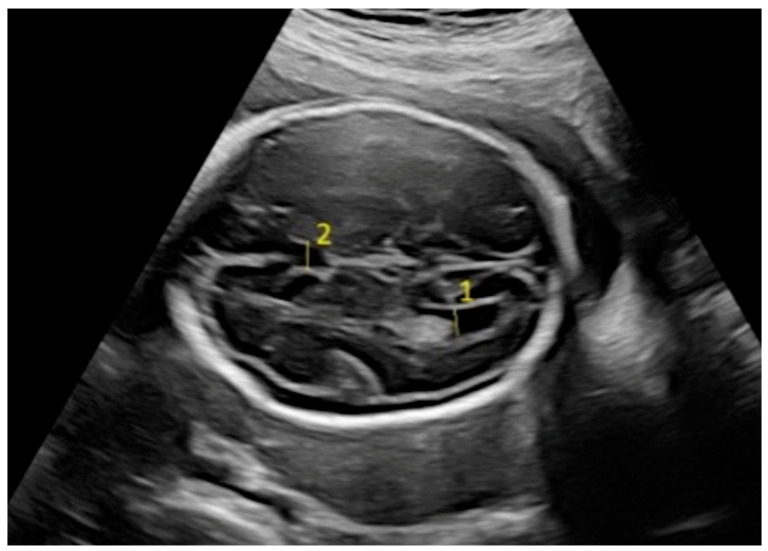
Fetal intracranial structures in the transventricular plane (1: posterior lateral ventricle; 2: cavum septum pellucidum).

Fetal biometric parameters and fetal intracranial structures were assessed via ultrasonography. The following fetal intracranial measurements were included: posterior lateral ventricle (PLV), cavum septi pellucidum (CSP), thalamus, cisterna magna (CM), and transverse cerebellar diameter (TCD). Prenatal ultrasonographic examinations were performed using a Voluson E8 Expert system (GE Healthcare, Tiefenbach, Austria) equipped with a 2–9 MHz 2D curvilinear transducer. All ultrasound assessments were conducted once by the same perinatologist, following the guidelines of the International Society of Ultrasound in Obstetrics and Gynecology and the World Association of Perinatal Medicine [18,19]. The PLV was measured on an axial transventricular plane that displays the anterior and posterior horns of the lateral ventricles, at the level of the atrium, perpendicular to the long axis of the ventricle. Calipers were placed on the inner (echogenic) edges of the ventricle walls at the widest point (inner-to-inner measurement). The thalamus, cerebellum, and CM were visualized on a transcerebellar plane. The transverse thalamic diameter was measured between the widest points of the thalami. For the TCD, calipers were placed on the outer edges of the cerebellum. CM was measured as the greatest distance between the posterior edge of the cerebellar vermis and the inner surface of the occipital bone. CSP was assessed on both the transventricular and transcerebellar planes, with the measurement taken on the transventricular plane. CSP width was measured from inner edge to inner edge, perpendicular to the midline (Figure 1 and Figure 2).

### Statistical Analysis

All statistical analyses were performed using the IBM^®^ SPSS^®^ Statistics 26 software package (IBM Corp., Armonk, NY, USA). Descriptive statistics are presented as the mean ± standard deviation, frequency, and percentage. Pearson’s chi-square test was used to analyze categorical variables. A one-way ANOVA was employed for comparisons among groups, and when significant differences were identified, post hoc Bonferroni tests were used for pairwise comparisons. A *p*-value less than 0.05 was considered statistically significant.

## 3. Results

A total of 52 pregnant women [hypothyroidism and Ab(+)] with hypothyroidism due to Hashimoto’s thyroiditis diagnosed before pregnancy who were receiving levothyroxine therapy and tested positive for TPOAb and TGAb, 51 pregnant women [hypothyroidism and Ab(−)] with no prior history of hypothyroidism but with negative TPOAb and TGAb levels and a first-trimester TSH > 2.5 mIU/L who received levothyroxine therapy during pregnancy, and 50 pregnant women [control] in the control group were included in the study.

According to the comparison among the study groups (Table 1), age, gestational age, gravidity, and parity were similar across the three groups (*p* > 0.05 for all). However, a significant difference was found in the body mass index (BMI) parameter (*p* = 0.012). A post hoc Bonferroni analysis was performed to determine which groups differed, indicating that both the hypothyroidism and Ab(−) and hypothyroidism and Ab(+) groups differed from the control group (*p* = 0.031 and *p* = 0.028, respectively).

Table 2 shows the results of the comparison of obstetric parameters among the groups. No statistically significant differences were observed in biparietal diameter (BPD), head circumference (HC), abdominal circumference (AC), femur length (FL), CSP, cerebellum, or CM measurements across the groups. Significant differences were observed in the PLV and thalamus parameters. For the PLV measurements, a significant difference was found only between the control group (6.48 ± 0.84 mm) and the hypothyroidism and Ab(+) group (5.92 ± 1.03 mm), with lower values in the hypothyroidism and Ab(+) group (*p* = 0.012). Similarly, for the thalamus measurements, a significant difference was noted only between the control group (18.20 ± 3.24 mm) and the hypothyroidism and Ab(+) group (16.71 ± 2.86 mm), again indicating lower values in the hypothyroidism and Ab(+) group (*p* = 0.027). These findings suggest that maternal hypothyroidism accompanied by thyroid autoantibodies may be associated with subtle alterations in specific fetal brain structures, particularly the PLV and thalamus, during mid-gestation.

BMI groups were subsequently formed based on the World Health Organization anthropometric scale, and comparisons were made among the study groups. A significant difference in the prevalence of being overweight or obese was noted between the hypothyroidism and Ab(+) group and the control group (67.3% vs. 48.0%, *p* = 0.002). These findings suggest that there may be an association between maternal hypothyroidism and an increased BMI, especially in the presence of thyroid autoantibodies. Comparison of BMI groups among the study groups is presented in Table 3.

Table 4 presents comparisons restricted to the overweight and obese BMI groups; no significant differences were found among the study groups in the BPD, HC, FL, PLV, CSP, cerebellum, or CM. However, significant differences were detected in the AC and thalamus measurements (*p* = 0.034 and *p* = 0.024, respectively). Post hoc analyses indicated that for the AC, there was a significant difference between the control and hypothyroidism and Ab(−) groups (*p* = 0.029). Regarding the thalamus parameter, significant differences were found between the control and hypothyroidism and Ab(+) groups, and also between the hypothyroidism and Ab(−) and hypothyroidism and Ab(+) groups (*p* = 0.022).

Finally, Table 5 shows comparisons restricted to the underweight and normal BMI groups; no significant differences were observed among the study groups in terms of the BPD, HC, AC, FL, CSP, thalamus, cerebellum, or CM. A significant difference was identified only in the PLV measurements (*p* = 0.044). Post hoc analysis revealed that the difference was between the control group and the hypothyroidism and Ab(+) group, with lower values detected in the hypothyroidism and Ab(+) group (*p* = 0.043).

## 4. Discussion

The CSP, PLV, CM, TCD, and thalamus are parameters that are often evaluated in fetal neurosonography. In this study, we found significant differences in fetal PLV and thalamus measurements among pregnant women who tested positive for TPOAb and TGAb and received levothyroxine therapy for hypothyroidism secondary to Hashimoto’s thyroiditis (hypothyroidism and Ab(+)). Compared with the control group, the hypothyroidism and Ab(+) group exhibited significantly lower PLV values (*p* = 0.012). Similarly, thalamus measurements were significantly lower in the hypothyroidism and Ab(+) group than in the control group (*p* = 0.027). These findings suggest that hypothyroidism and the presence of thyroid autoantibodies may lead to certain alterations in fetal brain structures. In particular, changes in critical brain regions such as the thalamus and PLV indicate that hypothyroidism may exert adverse effects on fetal neurological development.

The thalamus, located in the central part of the brain above the cerebral hemispheres, plays a key role in the processing and relay of sensory and motor signals. It has extensive functions, including sensory integration, motor coordination, cognitive processes, regulation of sleep and wakefulness, and emotion processing through its connections with the limbic system [20,21]. Any damage or dysfunction in the thalamus may have wide-ranging effects on sensory processing, motor control, and cognitive functions.

Because the fetal thyroid gland becomes functionally mature only after the 20th week of gestation, thyroid hormones transferred transplacentally from the mother before and after the onset of fetal thyroid function are crucial [22,23]. During the second trimester when neuronal proliferation, migration, and structural organization occur, fetal brain development largely depends on maternally derived thyroid hormones. In later stages of fetal brain development (glial cell proliferation, migration, and myelination) from the third trimester onward, the primary source of thyroid hormones is the fetal thyroid gland itself. At birth, approximately 30% of the serum thyroxine (T4) measured in the cord blood is known to originate from the mother [24]. This observation strongly suggests that maternal T4 transfer via the placenta persists until birth. In this context, severe maternal hypothyroidism that emerges in the second trimester may lead to irreversible neurological defects, whereas hypothyroidism diagnosed later in pregnancy tends to be less severe and partially reversible in terms of fetal brain injury [25].

In our study, we measured fetal intracranial structures in pregnancies between 18- and 24-weeks’ gestation, during the period in which the fetus is largely dependent on maternal thyroid hormones to evaluate fetal brain development. Assessing maternal antibody status is important because subclinical hypothyroidism and positive TPOAb in pregnant women are associated with an increased risk of adverse pregnancy outcomes including miscarriage, preterm birth, perinatal death, and postpartum dysfunction, and these outcomes can occur at lower TSH values compared to in TPOAb-negative women [26,27]. In the American College of Obstetricians and Gynecologists (ACOG) guidelines on thyroid disease in pregnancy, women who were TPOAb-positive with TSH > 2.5 mU/L had a clearly elevated risk of pregnancy-related complications, whereas TPOAb-negative women did not consistently exhibit such risks until TSH values exceeded 5–10 mU/L [28].

Several studies have reported a link between maternal thyroid autoimmunity and impaired neurodevelopment in offspring [16,29,30,31,32,33]. Thyroid autoantibodies may have significant implications during pregnancy for both the mother and the fetus. Their potential influence on fetal brain development includes delayed neurological maturation, intellectual disability, and motor or behavioral issues. In addition, they may affect placental function and thereby influence fetal neurodevelopment [11,25,34,35]. A study by Haddow and colleagues highlighted that mild or subclinical maternal thyroid dysfunction during pregnancy may be associated with disturbances in the normal brain development of the child [11], illustrating the critical role of thyroid hormones in neurological maturation. Impaired fetal brain development may arise from a combination of factors linked to perinatal hypothyroidism, including low thyroid hormone levels that directly affect the developing brain, as well as indirect effects such as alterations in placental function. Wasserman and colleagues evaluated TPOAb levels in the third trimester of pregnancy and found that children of TPOAb-positive mothers had lower IQ scores compared with those of TPOAb-negative mothers [33]. Previous research, which reported a significant association between a high maternal TPOAb concentration in the third trimester and sensorineural hearing loss in children (prevalence OR 7.5, 95% CI 2.4–23.3), suggested that the lower IQ scores might be mediated by sensorineural hearing loss [16]. Additionally, a study by Wilson et al., investigating the association between maternal TPOAb status and neonatal brain weight, found that infants of TPOAb-positive mothers had smaller head circumferences and reduced brain weights (β = −407; standard error [SE] = 0.200; *p* < 0.05 and β = −10.307; SE = 5.001; *p* < 0.05, respectively) [36]. A commonly accepted normal limit for the PLV measurement is less than 10 mm. Values exceeding this threshold can suggest the possibility of hydrocephalus, chromosomal anomalies, intracranial hemorrhage, infections, or other causes of fetal brain abnormalities [37,38]. In our study, the hypothyroidism and Ab(+) group showed lower PLV measurements compared with the control group, although they were still within normal ranges. This decrease is not associated with any known poor prognosis and does not carry clinical management implications. Although the long-term neurodevelopmental negative outcomes of maternal hypothyroidism and thyroid autoantibody positivity are well known, sonographic findings that can predict these negative outcomes in the intrauterine period have not yet been reported. In our study, we found smaller PLV and thalamus sizes in hypothyroid and autoantibody-positive cases, but our data only included mid-trimester examinations, so no evidence could be provided showing the relationship between these findings and long-term outcomes.

We found a statistically significant difference in the BMI between the control group and both hypothyroid groups—those with negative antibodies (hypothyroidism and Ab(−)) and those with positive antibodies (hypothyroidism and Ab(+)) (*p* = 0.012). These results suggest that hypothyroidism and antibody positivity may affect the body mass index in pregnancy, possibly through metabolic alterations. Such findings emphasize the importance of considering these factors in clinical practice, especially during pregnancy management and follow-up of thyroid function. When examining only the overweight and obese BMI subgroups, the hypothyroidism and Ab(+) group again exhibited significantly lower thalamus values than the hypothyroidism and Ab(−) and control groups, suggesting that this difference in thalamus measurement is not driven by BMI status.

Overall, these results underscore the importance of closely monitoring and adequately treating hypothyroidism during pregnancy. Tailored interventions and follow-up strategies may be necessary to support fetal neurological development in women with thyroid dysfunction during pregnancy. Such findings could have practical implications in perinatology, particularly in the monitoring and management protocols for fetal health among pregnant women with thyroid disorders. Previous studies investigating the neurological development of children born to mothers with hypothyroidism and thyroid autoimmunity have mainly focused on the postnatal and childhood periods. To our knowledge, no study to date has specifically examined fetal neurodevelopment and intracranial changes in this population. Hence, our research serves as a valuable initial contribution to this field.

The primary limitation of our study is that the evaluations were limited to mid-gestation; therefore, it remains unknown as to what changes may occur in the third trimester and postpartum period. Moreover, we did not assess fetal cortical structures, and our study was conducted at a single center with a limited number of participants. Future multicenter studies with different populations to assess fetal brain structures, including cortical structures, and to examine the relationship of findings to long-term postnatal outcomes would be useful to better understand the clinical impact of these findings.

In conclusion, this study was conducted to better understand the effects of hypothyroidism and thyroid autoimmunity on pregnancy. Our findings contribute to the current body of knowledge and highlight the need for future research aimed at refining management strategies for thyroid disorders in pregnancy.

## Figures and Tables

**Table 1:** Comparing parameters among groups.

Variables	Control (n = 50)	Hypothyroidism and Ab(−) (n = 51)	Hypothyroidism and Ab(+) (n = 52)	*p*-Value	Control vs. Hypothyroidism and Ab(−)	Control vs. Hypothyroidism and Ab(+)	Hypothyroidism and Ab(−) vs. Hypothyroidism and Ab(+)
	Mean ± SD				*p*-Value		
Age, years	29.20 ± 5.25	29.69 ± 5.14	30.08 ± 4.75	0.681	1.000	1.000	1.000
Gestational week	21.22 ± 1.63	21.43 ± 1.35	21.10 ± 1.45	0.510	1.000	1.000	0.755
Gravity	2.68 ± 1.54	2.39 ± 1.23	2.44 ± 1.38	0.541	0.899	1.000	1.000
Parity	1.22 ± 1.13	1.14 ± 1.06	1.00 ± 1.05	0.582	1.000	0.915	1.000
BMI, kg/m^2^	25.14 ± 4.33	27.67 ± 5.22	27.63 ± 4.87	0.012	0.031	0.028	1.000

One-way ANOVA analysis used and post hoc Bonferroni test used. *p* < 0.05 considered significant.

**Table 2:** Comparing sonographic parameters among groups.

Variables	Control (n = 50)	Hypothyroidism and Ab(−) (n = 51)	Hypothyroidism and Ab(+) (n = 52)	*p*-Value	Control vs. Hypothyroidism and Ab(−)	Control vs. Hypothyroidism and Ab(+)	Hypothyroidism and Ab(−) vs. Hypothyroidism and Ab(+)
	Mean ± SD				*p*-Value		
BPD	48.73 ± 5.29	49.35 ± 5.04	47.68 ± 8.38	0.415	1.000	1.000	0.571
HC	182.24 ± 17.93	185.64 ± 18.33	181.75 ± 19.75	0.521	1.000	1.000	0.880
AC	153.19 ± 28.38	159.10 ± 17.54	157.39 ± 19.39	0.390	0.548	1.000	1.000
FL	34.07 ± 4.91	34.72 ± 3.96	34.13 ± 5.18	0.747	1.000	1.000	1.000
PLV	6.48 ± 0.84	6.35 ± 0.98	5.92 ± 1.03	0.010	1.000	0.012	0.077
CSP	3.79 ± 0.61	3.74 ± 0.77	3.53 ± 0.72	0.127	1.000	0.170	0.363
Thalamus	18.20 ± 3.24	17.76 ± 2.38	16.71 ± 2.86	0.026	1.000	0.027	0.187
Cerebellum	21.48 ± 1.90	21.60 ± 2.18	21.34 ± 2.71	0.849	1.000	1.000	1.000
CM	4.86 ± 1.46	4.90 ± 1.39	4.81 ± 1.59	0.950	1.000	1.000	1.000

One-way ANOVA analysis used and post hoc Bonferroni test used. *p* < 0.05 considered significant. All units in the parameters are in mm.

**Table 3:** Comparing BMI groups among research groups.

BMI Groups	Groups			Total	*p*-Value	Control vs. Hypothyroidism and Ab(−)	Control vs. Hypothyroidism and Ab(+)	Hypothyroidism and Ab(−) vs. Hypothyroidism and Ab(+)
	Control	Hypothyroidism and Ab(−)	Hypothyroidism and Ab(+)			*p*-Value		
Underweight (<18.5 kg/m^2^)	1 (2.0)	1 (2.0)	0 (0)	2 (1.3)	0.249	0.231	0.002	0.519
Normal weight (18.5–25.0 kg/m^2^)	25 (50.0)	17 (33.3)	17 (32.7)	59 (38.6)				
Overweight and Obese (>25.0 kg/m^2^)	24 (48.0)	33 (64.7)	35 (67.3) *	92 (60.1)				
Total	50 (100)	51 (100)	52 (100)	153 (100)				

Pearson’s and Fisher’s exact chi-square test used. * *p* < 0.05 considered significant.

**Table 4:** Comparing obstetric parameters among groups (only overweight and obese BMI).

Variables	Control (n = 24)	Hypothyroidism and Ab(−) (n = 33)	Hypothyroidism and Ab(+) (n = 35)	*p*	Control vs. Hypothyroidism and Ab(−)	Control vs. Hypothyroidism and Ab(+)	Hypothyroidism and Ab(−) vs. Hypothyroidism and Ab(+)
	Mean ± SD				*p*		
BPD	48.25 ± 5.89	50.37 ± 4.85	47.49 ± 9.74	0.255	0.851	1.000	0.323
HC	181.47 ± 21.05	189.27 ± 17.71	182.59 ± 20.67	0.252	0.434	1.000	0.502
AC	145.58 ± 35.93	162.81 ± 15.76	156.96 ± 20.97	0.034	0.029	0.242	0.971
FL	33.81 ± 5.95	35.47 ± 3.70	34.25 ± 5.40	0.418	0.660	1.000	0.953
PLV	6.39 ± 0.85	6.28 ± 0.98	5.94 ± 1.13	0.200	1.000	0.300	0.509
CSP	3.87 ± 0.67	3.75 ± 0.80	3.57 ± 0.78	0.314	1.000	0.421	0.983
Thalamus	18.39 ± 2.96	18.06 ± 1.89	16.70 ± 2.93	0.024	0.786	0.018	0.022
Cerebellum	21.36 ± 2.03	21.99 ± 2.02	21.27 ± 2.82	0.238	0.954	1.000	0.631
CM	5.04 ± 1.40	4.95 ± 1.49	5.07 ± 1.62	0.925	1.000	1.000	1.000

One-way ANOVA analysis used and post hoc Bonferroni test used. *p* < 0.05 considered significant. All units in the parameters are in mm. BPD; biparietal diameter, HC; head circumference, AC; abdominal circumference, FL; femur length.

**Table 5:** Comparing obstetric parameters among groups (only underweight and normal BMI).

Variables	Control (n = 26)	Hypothyroidism and Ab(−) (n = 18)	Hypothyroidism and Ab(+) (n = 17)	*p*	Control vs. Hypothyroidism and Ab(−)	Control vs. Hypothyroidism and Ab(+)	Hypothyroidism and Ab(−) vs. Hypothyroidism and Ab(+)
	Mean ± SD				*p*		
BPD	49.17 ± 4.74	47.48 ± 4.98	48.08 ± 7.72	0.503	0.772	1.000	1.000
HC	182.96 ± 14.87	178.99 ± 18.03	180.03 ± 18.20	0.717	1.000	1.000	1.000
AC	160.21 ± 16.85	152.31 ± 19.02	158.28 ± 16.20	0.327	0.428	1.000	0.939
FL	34.31 ± 3.82	33.33 ± 4.14	33.88 ± 4.84	0.750	1.000	1.000	1.000
PLV	6.55 ± 0.85	6.46 ± 0.99	5.87 ± 0.80	0.044	1.000	0.043	0.303
CSP	3.72 ± 0.54	3.71 ± 0.72	3.43 ± 0.57	0.262	1.000	0.405	0.503
Thalamus	18.03 ± 3.54	17.22 ± 3.07	16.72 ± 2.79	0.415	1.000	0.600	1.000
Cerebellum	21.60 ± 1.80	20.86 ± 2.32	21.47 ± 2.56	0.529	0.828	1.000	1.000
CM	4.70 ± 1.52	4.80 ± 1.20	4.27 ± 1.40	0.490	1.000	0.991	0.798

One-way ANOVA analysis used and post hoc Bonferroni test used. *p* < 0.05 considered significant. All units in the parameters are in mm. BPD; biparietal diameter, HC; head circumference, AC; abdominal circumference, FL; femur length, PLV; posterior lateral ventricle, CSP; cavum septi pellucidum, CM; cisterna magna.

## Data Availability

The datasets analyzed in this study are available from the corresponding author upon reasonable request and with permission of the local Ethics Committee.
